# Mechanisms of *Gynostemma pentaphyllum* against non‐alcoholic fibre liver disease based on network pharmacology and molecular docking

**DOI:** 10.1111/jcmm.17410

**Published:** 2022-06-03

**Authors:** Cunzhi Wang, Pengrui Wang, Wenbin Chen, Yanyan Bai

**Affiliations:** ^1^ Shandong Provincial Hospital Affiliated to Shandong First Medical University Jinan Shandong China; ^2^ Shandong Provincial Hospital, Shandong University Jinan Shandong China

**Keywords:** *Gynostemma pentaphyllum*, molecular docking, network pharmacology, non‐alcoholic fibre liver disease

## Abstract

As a progressive chronic disease, the effective treatment for non‐alcoholic fibre liver disease (NAFLD) has not yet been thoroughly explored at the moment. The widespread use of *Gynostemma pentaphyllum* (Thunb) for its anti‐insulin resistance effect indicates that potential therapeutic value may be found in Thunb for NAFLD. Hence, this research aims to discover the latent mechanism of Thunb for NAFLD treatment. To achieve the goal of discovering the latent mechanism of Thunb for NAFLD treatment, molecular docking strategy integrated a network phamacology was adopted in the exploration. We acquire Thunb compounds with activeness from TCMSP database. We collect the putative targets of Thunb and NAFLD to generate the network. Key targets and mechanism are screened by PPI analysis, GO and KEGG pathway enrichment analyses. Molecular docking simulation is introduced into the study as assessment method. Through network analysis and virtual screening based on molecular docking, 2 targets (AKT 1 and GSK3B) are identified as key therapeutic targets with satisfying binding affinity. Main mechanism is believed to be the biological process and pathway related to insulin resistance according to the enrichment analyses outcomes. Particularly, the P13K–AKT signalling pathway is recognized as a key pathway of the mechanism. In conclusion, the study shows that Thunb could be a potential treatment against NAFLD and may suppress insulin resistance through the P13K–AKT signalling pathway. The result of the exploration provides a novel perspective for approaching experimental exploration.

## 1 INTRODUCTION

Non‐alcoholic fibre liver disease (NAFLD) means a series of illnesses from simple steatosis to non‐alcoholic steatohepatitis (NASH) and cirrhosis, which has been thought to be a hepatic indication for metabolic syndromes. It is currently recognized as a commonly seen long‐lasting and recurrent hepatic illness almost throughout the world, which is also most common for westerners.^1^ NAFLD has been proven by increasing studies as an indication of metabolic syndrome, with insulin resistance, hyperglycaemia and dyslipidaemia, and oxidative stress.^2^, ^3^ Insulin resistance may be a commonly seen pathogenic event.^4^ According to the ‘parallel multiple‐hit theory’,^5^ IR (insulin resistance), as an independent risk for NAFLD severity,^6^ is thought to be a ‘first blow’ triggering the illness, resulting in elevation of free fatty acids (FFAs) in hepatocytes.

In China, nowadays, the traditional Chinese medicine (TCM) has been made into practice for centuries. Increasing attention to it has been attracted because of its great effectiveness and minimal side effect upon preventing and treating metabolic disturbance, particularly in NAFLD.^8^, ^9^ *Gynostemma pentaphyllum* (Thunb), a plant belonging to the Cucurbitaceae family, has been mentioned in the books to have various therapeutic effects.^7^ In recent years, people have made systematic research on the chemical composition, pharmacology, clinical and health care development of Thunb. Thus far, acquisition of about 180 gypenosides is achieved out of G. pentaphyllum through isolation.^10^ In our previous studies, Thunb has been found to reduce insulin resistance in high‐sugar mice;^11^ however, Thunb fails to get completely understood from its chemical composition and function mechanisms, so it is warranted to explore the potential mechanism of Thunb to improve insulin resistance in NAFLD.

NAFLD is regulated by multiple genes or targets. For single target highly selective ligands drugs often do not change the overall state of the disease, it is difficult to effectively control or prevent the development of disease; therefore, the outcomes of the clinical trials were not satisfactory. Morphy et al. provide a new idea for drug discovery: multi‐target therapy.^12^ Exploration of drugs from multiple targets and multiple pathways offers an insight to develop new drugs. Network pharmacology functions as a big data integration approach upon the basis of large database resources and static algorithms, which is utilized for observing the interaction of multiple drug components, targets and disease mechanisms. It is also utilized for the prospective analysis of traditional Chinese medicine.^12^, ^13^, ^14^ Consequently, adoption of the network pharmacology method is conducted for investigating the latent therapeutic targets and pathways of Thunb during NAFLD treatment. NASH is a key process in the progression of NAFLD, so we also created a network to investigate the latent therapeutic targets and pathways of Thunb during NAFLD treatment. Molecular docking explorations are also done for further predicting the identification and interactive patterns amid Thunb as well as the targets predicted.

## 2 MATERIALS AND METHODS

### 2.1 Composite components of Thunb

The data of Thunb are mainly from a verified target databases: (1) pharmacology database of traditional Chinese medicine system^15^ (TCMSP, http://lsp.nwu.edu.cn/tcmsp.php).

### 2.2 Screening of bioactive components

In the clinical application of Thunb, oral administration has been normally utilized in Chinese medicine. Therefore, these two ADME models of oral bioavailability (OB^16^) and drug similarity (DL^17^) become the primary variables producing impact upon drug absorption in the gastrointestinal tract.^18^ Hence, OB≥30% and DL≥0.18 should be employed as screening conditions for screening the bioactive components of Thunb. (OB works as an important index for the objective assessment of the oral bioavailability of drug molecules, while DL is an index for the assessment of drug molecular drugging).

### 2.3 Prediction of Thunb action target

Currently, there are many methods to predict drug targets, according to its prediction principle, classification of those approaches into four types is performed:(1) Structure‐based prediction approaches, which are based on the structural properties of compounds; (2) Molecular Activity‐based prediction approaches, which are upon the basis of the protein interaction network (PIN); (3) Side‐effect‐based prediction approaches upon the basis of the adverse reactions; (4) Multi‐Omics‐based predictions methods, upon the basis of the predicting theory that identical medication has action upon those identical targets.^17^ The exploration used TCMSP^15^ (http://lsp.nwu.edu.cn/tcmsp.php) and SymMap^19^ (https://www.symmap.org/), to predict these targets of Thunb components. Considering that every interaction put forward should possess traceability to the original information sources in this database.

### 2.4 NAFLD‐related targets

Depending on the features of every database, this exploration chose to use distinctive keywords and standards to retrieve NAFLD‐related targets. ‘non‐alcoholic fibre liver disease/ non‐alcoholic steatohepatitis’ should be utilized to be a keyword in searching the Drugbank database^20^ (http://www.drugbank.ca). ‘Non‐alcoholic fibre liver disease/ non‐alcoholic steatohepatitis’ should be utilized to be a keyword in searching the GeneCards^21^ (http://www.genecards.org/).

### 2.5 Protein–protein interaction analysis

The normal life activities of cells are inseparable from the interaction of proteins and other molecules, like genes, small molecule compounds and other proteins. A complete organism interaction set can be shown through a PPI network, in which a single protein is thought to be a node, while a pair of proteins have a physical interaction as an edge.^22^ On the String^23^ (https://string‐db.org/), the identifiers meant ‘Homo sapiens, multiple proteins’; the data sources of PPI were chosen. After that, construction of PPI networks for targets in relation to NAFLD/NASH and Thunb was done. Identification of essential targets in anti‐NAFLD and anti‐NASH system should be performed through utilization of network data for topology features analyses.

### 2.6 Gene ontology and KEGG analysis

As a reference knowledge base, KEGG can be utilized to interpret massive molecular data sets from biology, like genome and metagenome sequences. It has accumulated experimental knowledge of advanced functions of cells and organisms represented by KEGG molecular network, comprising KEGG pathway diagram, Brite hierarchy and KEGG module.^24^ For revealing the latent mechanism of Thunb treatment for NAFLD and NASH, the overlapping targets had been introduced into the Database for Integrated Discovery, Visualization and Annotation, DAVID 6.8^25^ (http://david.ncifcrf.gov/), for performing KEGG pathway analyses and GO functional analyses. Functional annotation of enrichment was performed by Benjamini correction (P < 0.05). KEGG pathway enrichment maps and GO enrichment were performed under R version 3.2.0.

### 2.7 Network construction

Using Cytoscape3.6.1^26^ (http://cytoscape.org) as the biomolecular interaction network integration model of software environment, the following were built:(1) the compound‐target network; (2) PPI network of compound‐NAFLD/compound‐NASH target; and (3) compound‐target‐pathway network.

Three metrics had been utilized for evaluating the topology features for every node. (1) ‘Betweenness Centrality’: The ratio of nodes has the shortest path through the network,^27^ (2) ‘Closeness Centrality’ serves as a reflection on the distance amid one node and another ones^28^ (3) ‘Degree’ means the quantity of nodes that have direct relation with these nodes within this network.^29^ Level of the three parameters gives a reflection on the significance of those nodes within the network topology, the higher the value node, the more important.

### 2.8 Molecular docking simulation

#### 2.8.1 Target protein preparation

Downloading of the crystal structures for hub genes was made out of RCSB Protein Data Bank^30^ (http://www.pdb.org/). Modification of those complexes already downloaded was done through utilizing the AutoDock Tools 1.5.6^31^ (http://mgltools.scripps.edu/documentation/links/autodock) for removing the water molecules, which had been utilized for preparing receptors, comprising the addition of hydrogen and set‐up of docking paraments. Upon maximizing the settings, ‘Grid box’ had been utilized for performing the subsequent docking.

#### 2.8.2 Ligand preparation

Prior to docking, we downloaded the 3D structure of Thunb from TCMSP (https://tcmspw.com/tcmsp.php) and applied minimum energy to do subsequent docking.

#### 2.8.3 Molecular docking

We saved every ligand and receptor file in pdbqt format. After that, Autodock Vina was utilized,^32^ which is an open‐source package with great availability for free, for evaluating and verifying the binding affinity for compound–target relation. With utilization of PyMol2.3.0 software, visualization of the binding models was realized.

## 3 RESULTS

### 3.1 Screening of Thunb active ingredients

In line with the above retrieval standards (OL≥30% DL≥0.18), altogether, 24 active constituents had been screened, and Table 1 below reveals their basic information.

**TABLE 1:** Screening results of gynostemma pentaphyllum active constituents

MolID	MolName	OB (%)	DL
MOL000338	3′‐methyleriodictyol	51.61	0.27
MOL000351	Rhamnazin	47.14	0.34
MOL000359	Sitosterol	36.91	0.75
MOL004350	Ruvoside_qt	36.12	0.76
MOL004355	Spinasterol	42.98	0.76
MOL005438	Campesterol	37.58	0.71
MOL005440	Isofucosterol	43.78	0.76
MOL007475	Ginsenoside f2	36.43	0.25
MOL000953	CLR	37.87	0.68
MOL000098	Quercetin	46.43	0.28
MOL009855	(24S)‐Ethylcholesta‐5,22,25‐trans‐3beta‐ol	46.91	0.76
MOL009867	4α,14α‐dimethyl‐5α‐ergosta‐7,9(11),24(28)‐trien‐3β‐ol	46.29	0.76
MOL009877	cucurbita‐5,24‐dienol	44.02	0.74
MOL009878	Cyclobuxine	84.48	0.7
MOL009888	Gypenoside XXXVI_qt	37.85	0.78
MOL009928	Gypenoside LXXIV	34.21	0.24
MOL009929	Gypenoside LXXIX	37.75	0.25
MOL009938	Gypenoside XII	36.43	0.25
MOL009943	Gypenoside XL	30.89	0.21
MOL009969	Gypenoside XXXV_qt	37.73	0.78
MOL009971	Gypenoside XXVII_qt	30.21	0.74
MOL009973	Gypenoside XXVIII_qt	32.08	0.74
MOL009976	Gypenoside XXXII	34.24	0.25
MOL009986	Gypentonoside A_qt	36.13	0.8

### 3.2 NAFLD target network

**FIGURE 1:**
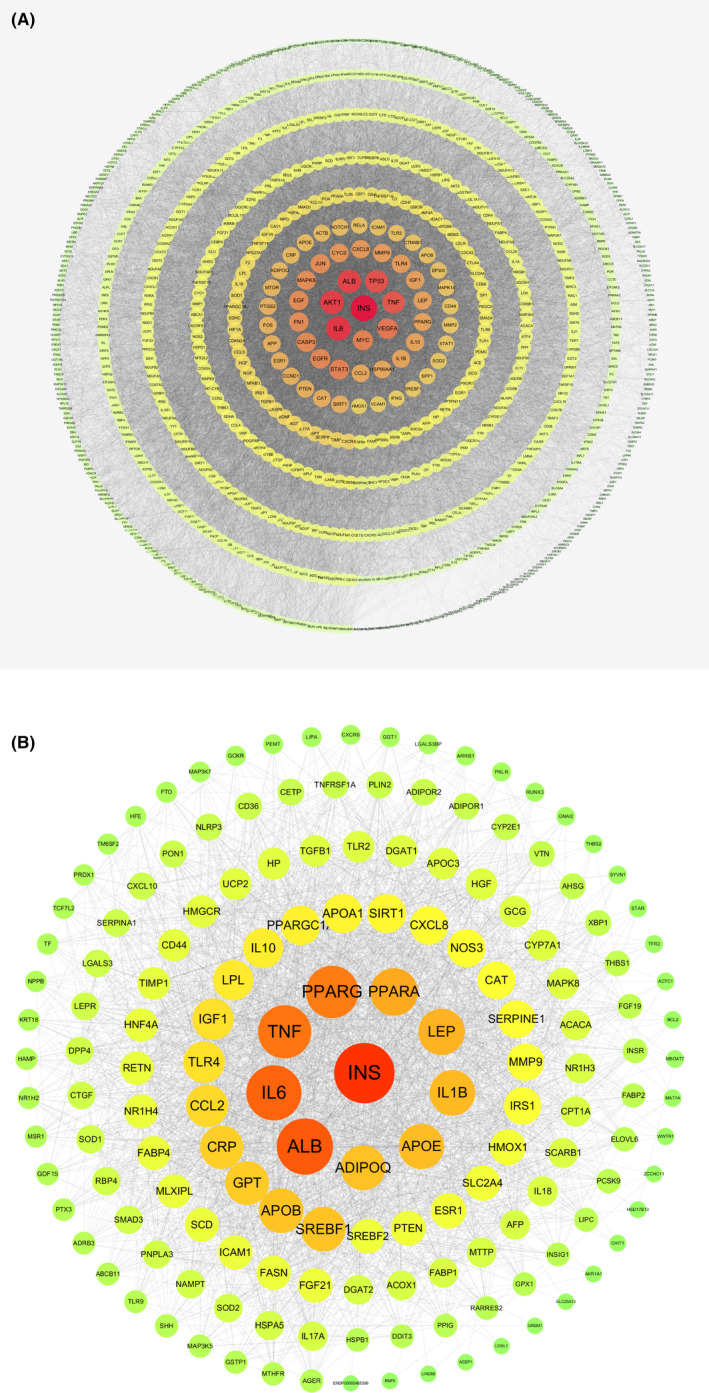
(A) PPI network of NAFLD targets. (B) PPI network of NASH targets. The nodes range in size and colour from largeness to smallness, greenness to redness, according to descending sequence of extent

According to Figure 1A, the PPI network gave a reflection on the interactive statuses amid the total of 937 targets, which had been recorded as the disease potential targets of NAFLD. As for the node degree, six targets were highly correlated with the pathological process of NAFLD, including INS (insulin, degree = 453), IL6 (interleukin‐6, degree = 400), AKT1 (RAC‐alpha serine/threonine‐protein kinase, degree = 382), ALB (albumin, degree = 375), TP53 (tumour protein p53, degree = 349) and TNF (tumour necrosis factor, degree =339). In addition according to relevance score >0.5, there is the total of 208 targets had been recorded as the disease potential targets of NASH.

### 3.3 PPI network of compound‐NAFLD/compound‐NASH targets

**FIGURE 2:**
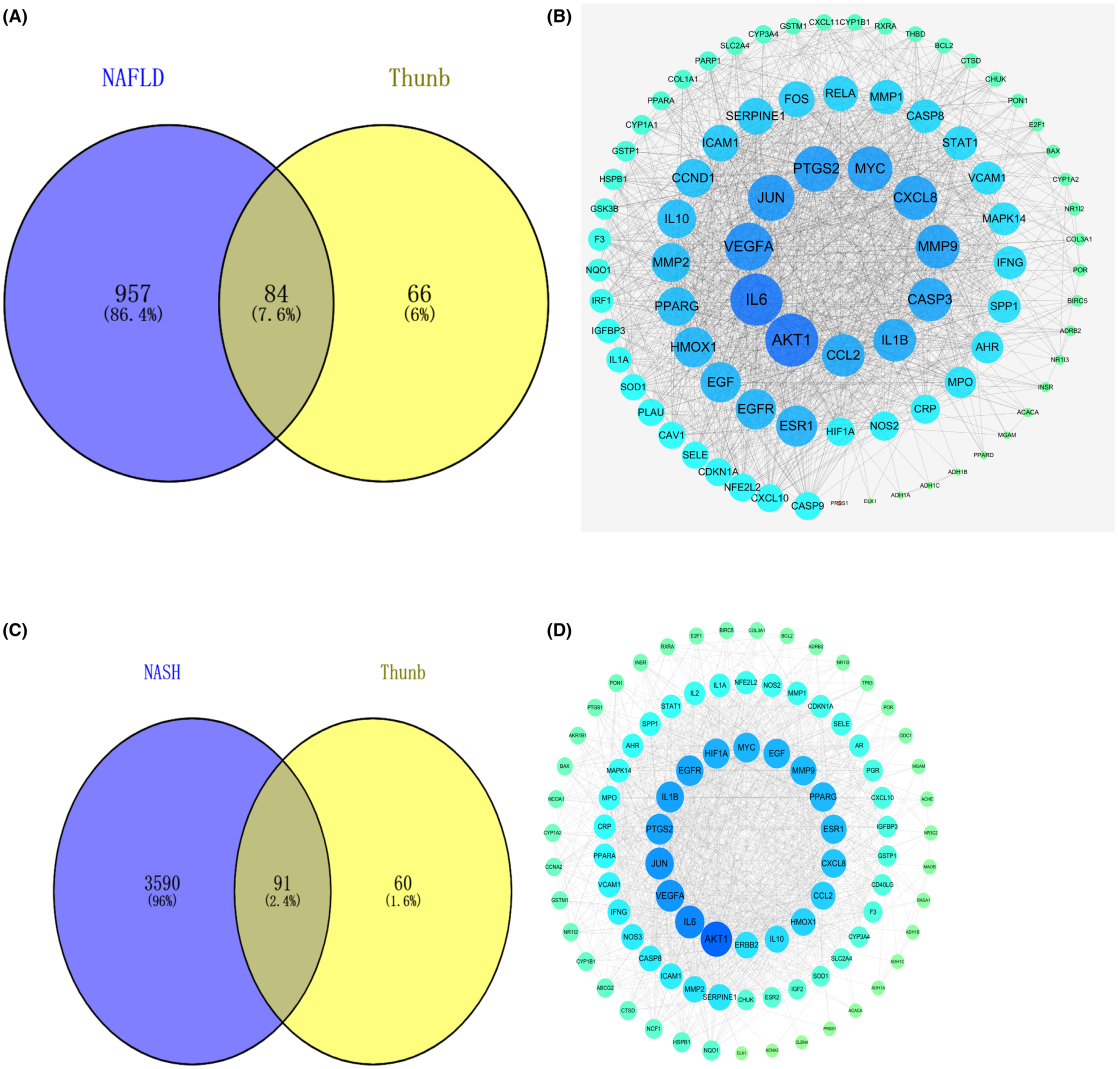
Venn diagram and PPI network of compound‐NAFLD targets. (A) Venn diagram of intersecting targets of Thunb and NAFLD. (B) PPI network of compound‐NAFLD targets. (C) Venn diagram of intersecting targets of Thunb and NASH. (D) PPI network of compound‐NASH targets. Those nodes vary in their dimension and colour, which are displayed according to descending sequence of degree values from largeness to smallness and greenness to blueness

Upon the basis of outcomes hereinabove, the 150 assumed targets of Thunb were mapped to 937 NAFLD‐related targets for obtaining overlapping targets. Thus, 84 targets had been confirmed to be candidates for the treatment of NAFLD (Figure 2A) and 91 targets had been confirmed to be candidates for the treatment of NASH. Then, a PPI network should be built up for evaluating the function of those targets among complex diseases and found that the interaction between them. The topology characteristics of PPI network had been analysed, and sortation of 84 targets into descending sequence was conducted. In Figure 2B, nodes according to the degree are arranged into concentric circles, centre of the innermost circle consists of 11 nodes, including AKT1, IL6 (interleukin‐6), VEGFA (vascular endothelial growth factor A), JUN (transcription factor AP‐1), PTGS2 (prostaglandin‐endoperoxide synthase 2), CXCL8 (interleukin‐8), MMP9 (matrix metallopeptidase 9), CASP3 (caspase‐3), IL1B (interleukin‐1 beta) and CCL2 (monocyte chemotactic protein [MCP]‐1).

### 3.4 GO and KEGG pathway analysis

**FIGURE 3:**
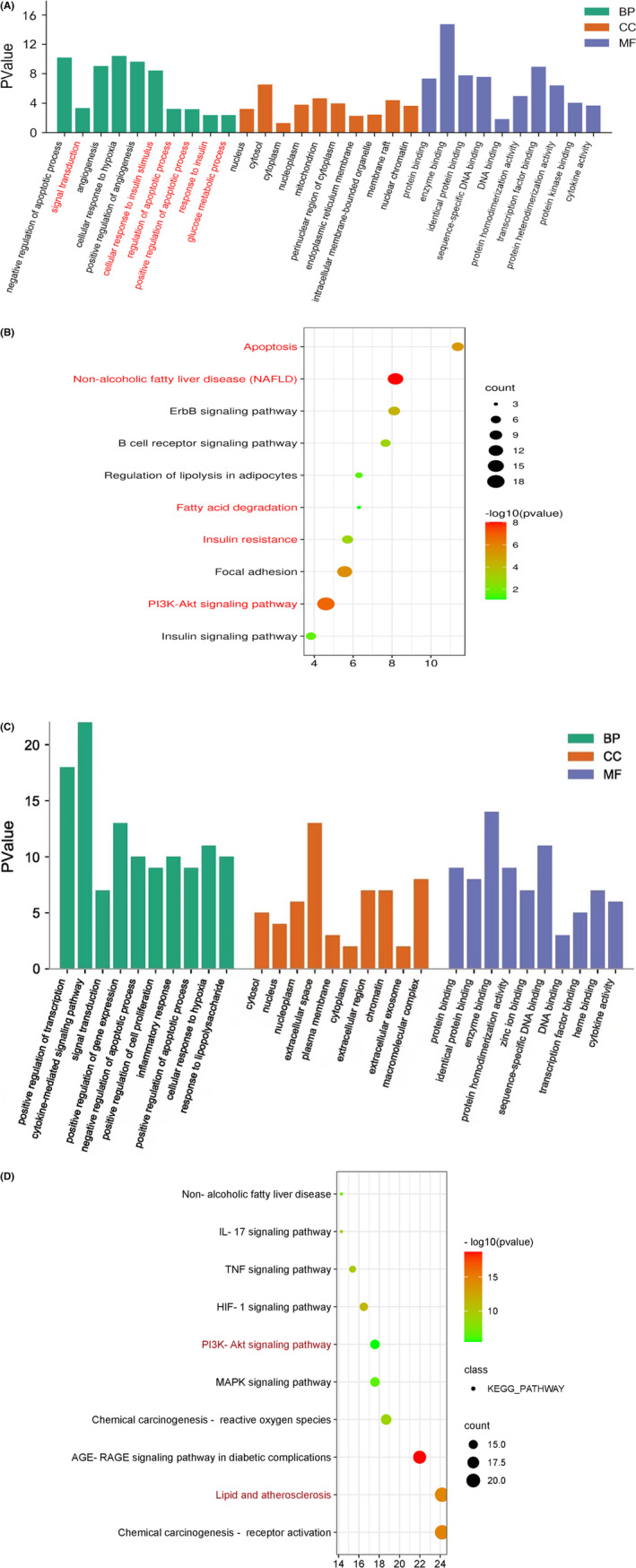
Enrichment analyses of potential targets. (A) GO enrichment analyses of compound‐NAFLD (C) GO enrichment analyses of compound‐NASH. BP. Biological processes, CC; cellular component, MF; molecular functionalities. The top 10 terms of each part are shown. (B) KEGG pathway analyses of compound‐NAFLD. (D) KEGG pathway analyses of compound‐NASH. The bubbles vary in their dimension, which are displayed from large to small according to descending sequence of the quantity of the latent targets related to pathways

For additionally expounding the mechanism of drug therapy in a systematical manner, utilization of DAVID had been done for performing enrichment analyses of genes. The top10 KEGG pathways and GO items were selected according to gene count and P value. According to the findings from biological processes, the targets were enriched in negatively regulated apoptotic process (GO:0043066), signal transduction (GO:0007165), positively regulated apoptotic process (GO:0043065), response to insulin (GO:0032868) and glucose metabolic process (GO:0006006) (Figure 3A,C). A total of 99 pathways were obtained by KEGG analyses, and majority got involvement with insulin resistance and apoptotic process‐related pathways like P13K–AKT signalling pathway (hsa04151), non‐alcoholic fatty liver disease (NAFLD) (hsa04932), insulin resistance (hsa04931) and apoptosis (hsa04210) (Figure 3B,D).

### 3.5 Molecular docking analysis

**FIGURE 4:**
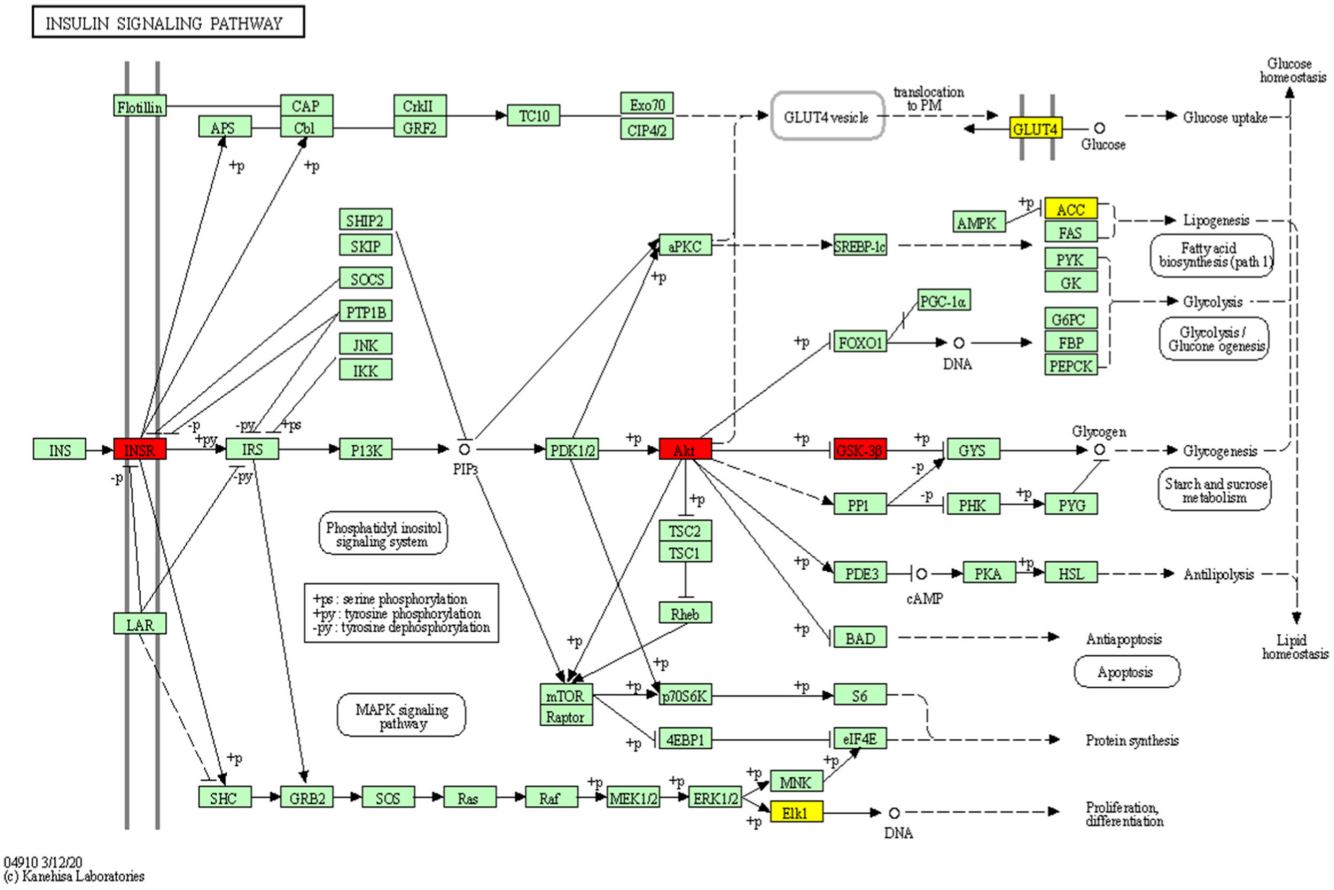
P13K–AKT signalling pathway has a central function in anti‐NAFLD system of Thunb. The yellow nodes refer to overlapping targets of NAFLD and Thunb targets, the red nodes represent key targets, and the green nodes mean the other targets in P13K–AKT signalling pathway

In this study, we investigated the potential interaction activities between 13 hub genes of Thunb and their relevant compounds by molecular docking verification. Meanwhile, the docking affinity values revealed by Autodock Vina^32^ were utilized for further screening the retrieved targets and active compounds. A total of 15 pairs were delivered to the docking simulation (Table 2). In the docking outcomes, a majority of the binding possessed great binding affinity, whose mean value was −5.97 kcal/mol. The modes of 3 binding complexes are shown in Figure 4, such as GSK3B‐Rhamnazin docking (−5.82 kcal/mol), AKT‐Quercetin docking (−6.36 kcal/mol) and INSR‐Quercetin docking (−6.85 kcal/mol). When the docking affinity possesses higher absolute values, a stronger binding capability was seen in these compounds to the target active site. Specifically, taking the INSR‐Quercetin docking as an example, small molecule ligand Quercetin may be suitable for the interface pocket under the formation of interacted amino acid residues in the protein (Figure 4). It revealed five hydrogen bonds were formed amid ligand and residues in MET 1079, ASP 1150 and LEU 1002. Five hydrogen bond interactions were 1.9, 2.0, 2.1, 2.1 and 2.2 A, respectively, which were considered as strong interactions. Overall, we found that different interactive forms would make a determination on the capability of binding affinity, and the hydrogen bond was the primary forms of interactive statuses for 3 docking complexes.

**TABLE 2:** Outcomes of 13 hub genes and compounds of Thunb molecular docking

Number	Hub genes	Uniprot ID	Compound	Docking affinity [kcal/mol]
1	AKT1	P31749	Quercetin	−6.36
2	IL6	P05231	Quercetin	−4.42
3	VEGFA	P15692	Quercetin	−6.89
4	JUN	P05412	Quercetin	−3.39
5	PTGS2	P35354	Quercetin	−6.95
			Rhamnazin	−6.73
			3‐methyleriodictyol	−6.34
6	MYC	P01106	Quercetin	−3.23
7	CXCL8	P10145	Quercetin	−7.57
8	MMP9	P14780	Quercetin	−6.22
9	CASP3	P42574	Quercetin	−5.88
10	IL1B	P01584	Quercetin	−6.52
11	CCL2	P13500	Quercetin	−6.41
12	INSR	P06213	Quercetin	−6.85
13	GSK3B	P49841	Rhamnazin	−5.82

## 4 DISCUSSION

Recently, an increasing quantity of explorations had been conducted in medication discovery and combination therapy of traditional Chinese medicine for complex diseases such as NAFLD, among which network pharmacological methods have played an important role. The multiply components and multiply target features of traditional Chinese medicine make it possible to control symptoms through different biological processes and effectively prevent the deterioration of the disease.^33^ In this exploration, construction of a network hereinbelow was done for revealing the latent targets and pathways of Thunb during NAFLD/NASH treatment.

Upon integration and consolidation of data out of multiple accessible databases, 4 active compounds of Thunb had action upon 84 different targets in association with NAFLD and 91 different targets in association with NASH. Our previous studies have shown that Thunb can reduce insulin resistance in NAFLD,^11^ however, its bioactive components and mechanisms of action have not been identified. Therefore, network pharmacology was utilized for screening the active components and targets of Thunb; the potential mechanism of Thunb was also acquired to reduce insulin resistance in NAFLD. The PPI network of NAFLD targets gave a reflection of the latent pathogenesis of NAFLD, indicating its consistency with previous research.

A widely reported viewpoint rests with that insulin resistance has a central function during those procedures through permitting too much fatty acid to flow into adipose tissue and impairing the disposal of peripheral glucose.^34^ FFAs are absorbed by the liver in proportion to their delivery rate and have a direct stimulation upon the synthesis of very low‐density lipoprotein (VLDL) in hepatocytes.^35^ When these FFAs get absorbed by hepatocytes, they will temporarily enter the storage pool prior to oxidizing or secreting in the form of VLDL.^36^ In addition, due to excessive carbohydrates, like fructose, another significant contributor to the hepatocyte triglyceride pool refers to de novo adipogenesis.^37^, ^38^ Three main sites of insulin resistance include the muscle, liver and adipose tissue. Insulin resistance has been considered to begin in muscle tissue, with inflammation alteration under the mediation of immune and excessive FFAs, resulting in ectopic lipid deposition.^39^, ^40^ Muscles account for 70% of glucose disposal. As muscle uptake is impaired, excessive glucose is back to the liver elevating de novo lipogenesis and circulating free fatty acids.^41^ When the delivery of these energy substrates exceeds the capacity of the liver to process them, the triglyceride pool will continue to grow. These FFA metabolites result in liver cell damage characterized by endoplasmic reticulum stress, apoptosis, inflammation, necrosis and deformities like balloon and Mallory–Denk body formation.^34^

**FIGURE 5:**
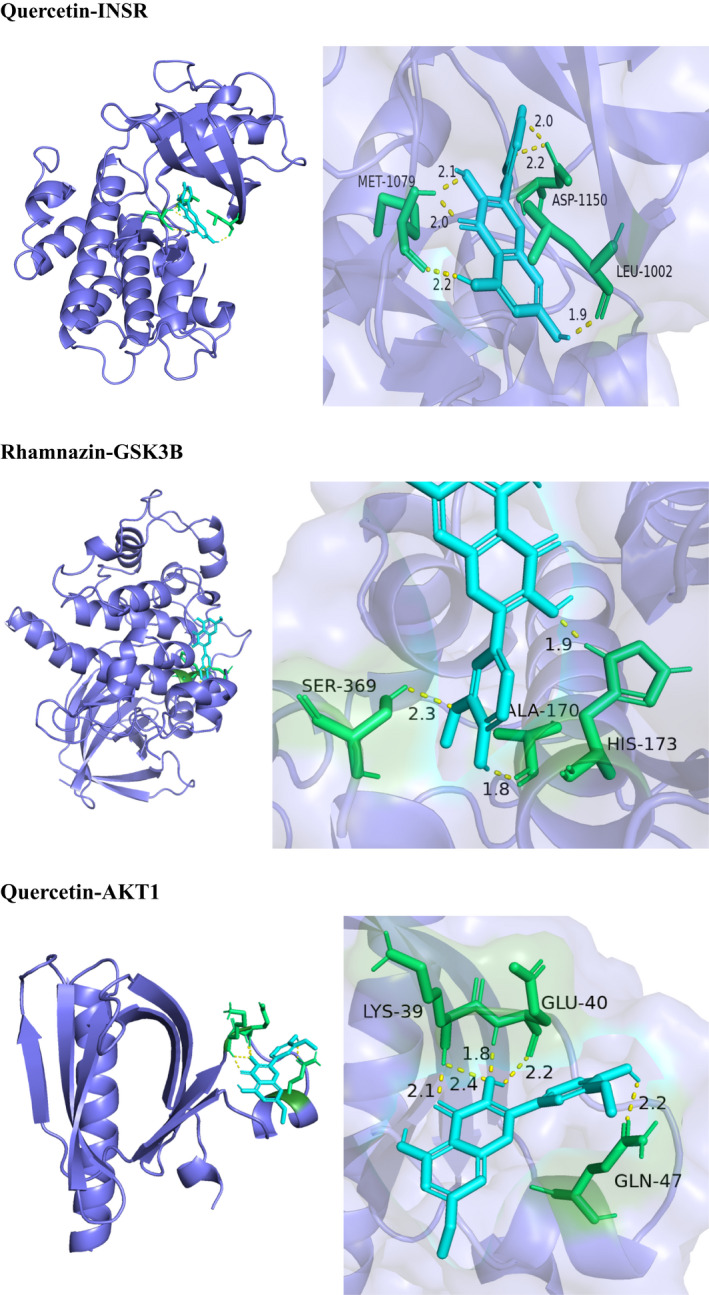
Molecular docking models of active compounds binding to potential targets. The 3 pairs of molecular docking simulation are revealed. Schematics (2D) displayed the interactive statuses amid compounds and residues surrounded. The yellow dashed lines mean hydrogen bonds and the interacted distances are presented beside to the bonds. Schematics (3D) reveal that molecular model of the compound is in the binding pocket of the protein. The compounds are presented as stick model with blue colour. The amino acid residues surrounding are displayed by surface

As is universally known, P13K/Akt/GSK3β pathway functions as one of the most important insulin signalling pathways. Massive proof indicated the direct increase in de novo lipogenesis by deregulated P13K/Akt/GSK3β signalling pathway and indirect elevation of FFA flux to the liver through the increase in adipose tissue lipolysis.^20^ (Figure 5) Meantime, molecular docking simulation amid 26 hub genes and 4 active compounds were conducted for complementing key targets. These outcomes revealed the good docking affinity for each target–compound pairs possess, particularly GSK‐3β and AKT1 (Figure 5).

Glycogen synthase kinase‐3 (GSK‐3) serves as a post‐insulin receptor signalling protein. There are two isomers, GSK‐3β and GSK‐3α,^42^ which are inactivated when phosphoric acid is added and activated when phosphoric acid is deactivated.^43^ Under normal circumstances, insulin inactivates GSK‐3β by phosphorylating it, and the inactivated GSK‐3β loses its inhibitory effect on liver glycogen, thus promoting the synthesis of liver glycogen.^44^ However, in type 2 diabetes mellitus, because of insulin resistance, the GSK‐3β phosphorylation pathway is blocked, liver glycogen synthesis is inhibited, and liver glycogen content decreases, resulting in increased blood sugar concentration.^20^ The liver P13K/AKT signalling pathway is the most important and classic pathway for the regulation of gsk‐3 activity.^21^ Under normal circumstances, after insulin binding to the insulin receptor, the insulin receptor substrates (IRSs) are phosphorylated and activate phosphatidylinositol‐3 kinase (PI3K) by binding to the Src homology 2 (SH2) domain. Activated PI3K phosphorylates phosphatidylinositol diphosphate (PIP2) to form phosphatidylinositol triphosphate (PIP3). PIP3 recruits and activates phosphatidylinositol‐dependent protein kinase 1 (PKD1) and protein kinase B (AKT), AKT inactivates glycogen synthase kinase‐3β (GSK‐3β) via Ser 9 phosphorylation. Heptic glycogen synthesis are increased and blood glucose level drops.^22^ According to this exploration, the PPI network and the result of KEGG showed the effect of Thunb on INSR/AKT/GSK‐3β. In addition, the results of compound‐NASH showed the importance of P13K–AKT pathway in NASH. Therefore, it can be summarized from the results that the P13K–AKT signalling pathway has an important function in NAFLD, and intervening the process might become a latent therapeutic target for Thunb against NAFLD.

## AUTHOR CONTRIBUTIONS

**Cunzhi Wang:** Conceptualization (lead); data curation (lead); formal analysis (lead); investigation (lead); methodology (lead); resources (lead); validation (lead); visualization (lead); writing – original draft (lead). **Wenbin Chen:** Methodology (equal); project administration (equal); supervision (equal); validation (equal). **Yanyan Bai:** Conceptualization (equal); project administration (equal); supervision (equal); writing – review and editing (equal). **Pengrui Wang:** Formal analysis (supporting); investigation (supporting); resources (supporting); software (supporting).

## CONFLICT OF INTEREST

The authors make a declaration that there is non‐existence of any competitive economic interests or personal relations amid themselves, because of which it will not produce any impact on the work involved in this article.

## Data Availability

The data that support the findings of this study are available from the corresponding author upon reasonable request.

## References

[1] Bessone F , Razori MV , Roma MG . Molecular pathways of nonalcoholic fatty liver disease development and progression. Cell Mol Life Sci. 2019;76(1):99‐128. doi:10.1007/s00018-018-2947-0 30343320 PMC11105781

[2] Zhang X , Asllanaj E , Amiri M , et al. Deciphering the role of epigenetic modifications in fatty liver disease: a systematic review. Eur J Clin Invest. 2020;22:e13479. doi:10.1111/eci.13479 PMC8243926 33350463

[3] Lonardo A , Byrne CD , Caldwell SH , Cortez‐Pinto H , Targher G . Global epidemiology of nonalcoholic fatty liver disease: meta‐analytic assessment of prevalence, incidence, and outcomes. Hepatology. 2016;64(4):1388‐1389. doi:10.1002/hep.28584 27038241

[4] Marchesini G , Marzocchi R , Agostini F , Bugianesi E . Nonalcoholic fatty liver disease and the metabolic syndrome. Curr Opin Lipidol. 2005;16(4):421‐427. 15990591 10.1097/01.mol.0000174153.53683.f2

[5] Tilg H , Moschen AR . Evolution of inflammation in nonalcoholic fatty liver disease: the multiple parallel hits hypothesis. Hepatology. 2010;52(5):1836‐1846. doi:10.1002/hep.24001 21038418

[6] Eguchi Y , Eguchi T , Mizuta T , et al. Visceral fat accumulation and insulin resistance are important factors in nonalcoholic fatty liver disease. J Gastroenterol. 2006;41(5):462‐469. doi:10.1007/s00535-006-1790-5 16799888

[7] Niu Y , Yan W , Lv J , Yao W , Yu L . 4.Characterization of a novel polysaccharide from tetraploid *Gynostemma pentaphyllum* Makino. J Agric Food Chem. 2013;61:4882‐4889. 23627413 10.1021/jf400236x

[8] Huang TH , Razmovski‐Naumovski V , Salam NK , et al. A novel LXR‐α activator identified from the natural product *Gynostemma pentaphyllum* . Biochem Pharmacol. 2005;70:1298‐1308. 16154115 10.1016/j.bcp.2005.07.033

[9] Circosta C , De Pasquale R , Occhiuto F . Cardiovascular effects of the aqueous extract of *Gynostemma pentaphyllum* Makino. Phytomedicine. 2005;12:638‐643. 16194050 10.1016/j.phymed.2004.06.023

[10] Hung TM , Thu CV , Cuong TD , et al. Dammarane type glycosides from *Gynostemma pentaphyllum* and their effects on IL‐4‐induced eotaxin expression in human bronchial epithelial cells. J Nat Prod. 2010;73:192‐196. 20104880 10.1021/np9006712

[11] Jia N , Lin X , Ma S , et al. Amelioration of hepatic steatosis is associated with modulation of gut microbiota and suppression of hepatic miR‐34a in *Gynostemma pentaphylla* (Thunb.) Makino treated mice. Nutr Metab (Lond). 2018;15:86. doi:10.1186/s12986-018-0323-6 30555521 PMC6282400

[12] Zhang R , Zhu X , Bai H , Ning K . Network pharmacology databases for traditional Chinese medicine: review and assessment. Front Pharmacol. 2019;21(10):123. doi:10.3389/fphar.2019.00123 PMC6393382 30846939

[13] Xu X , Zhang W , Huang C , et al. A novel chemometric method for the prediction of human oral bioavailability. Int J Mol Sci. 2012;13(6):6964‐6982. doi:10.3390/ijms13066964 22837674 PMC3397506

[14] Lyu M , Wang YF , Fan GW , Wang XY , Xu SY , Zhu Y . Balancing herbal medicine and functional food for prevention and treatment of cardiometabolic diseases through modulating gut microbiota. Front Microbiol. 2017;8(8):2146. doi:10.3389/fmicb.2017.02146 29167659 PMC5682319

[15] Ru J , Li P , Wang J , et al. TCMSP: a database of systems pharmacology for drug discovery from herbal medicines. J Chem. 2014;16(6):13. doi:10.1186/1758-2946-6-13 PMC4001360 24735618

[16] Feng W , Ao H , Yue S , Peng C . Systems pharmacology reveals the unique mechanism features of Shenzhu Capsule for treatment of ulcerative colitis in comparison with synthetic drugs. Sci Rep. 2018;8(1):16160. doi:10.1038/s41598-018-34509-1 30385774 PMC6212405

[17] Huang G , Yan F , Tan D . A review of computational methods for predicting drug targets. Curr Protein Pept Sci. 2018;19(6):562‐572. doi:10.2174/1389203718666161114113212 27842478

[18] Murakami Y , Tripathi LP , Prathipati P , Mizuguchi K . Network analysis and in silico prediction of protein‐protein interactions with applications in drug discovery. Curr Opin Struct Biol. 2017;44:134‐142. doi:10.1016/j.sbi.2017.02.005 28364585

[19] Wu Y , Zhang F , Yang K , et al. SymMap: an integrative database of traditional Chinese medicine enhanced by symptom mapping. Nucleic Acids Res. 2019;47(D1):D1110‐D1117. doi:10.1093/nar/gky1021 30380087 PMC6323958

[20] Probst D , Reymond JL . Exploring DrugBank in virtual reality chemical space. J Chem Inf Model. 2018;58(9):1731‐1735. doi:10.1021/acs.jcim.8b00402 30114367

[21] Rebhan M , Chalifa‐Caspi V , Prilusky J , Lancet D . GeneCards: a novel functional genomics compendium with automated data mining and query reformulation support. Bioinformatics. 1998;14(8):656‐664. doi:10.1093/bioinformatics/14.8.656 9789091

[22] Gupta SK , Srivastava M , Osmanoglu Ö , Dandekar T . Genome‐wide inference of the Camponotus floridanus protein‐protein interaction network using homologous mapping and interacting domain profile pairs. Sci Rep. 2020;10(1):2334. doi:10.1038/s41598-020-59344-1 32047225 PMC7012867

[23] Szklarczyk D , Morris JH , Cook H , et al. The STRING database in 2017: quality‐controlled protein‐protein association networks, made broadly accessible. Nucleic Acids Res. 2017;45(D1):D362‐D368. doi:10.1093/nar/gkw937 27924014 PMC5210637

[24] Kanehisa M , Furumichi M , Tanabe M , Sato Y , Morishima K . KEGG: new perspectives on genomes, pathways, diseases and drugs. Nucleic Acids Res. 2017;45(D1):D353‐D361. doi:10.1093/nar/gkw1092 27899662 PMC5210567

[25] Huang da W , Sherman BT , Lempicki RA . Systematic and integrative analysis of large gene lists using DAVID bioinformatics resources. Nat Protoc. 2009;4(1):44‐57. doi:10.1038/nprot.2008.211 19131956

[26] Shannon P , Markiel A , Ozier O , Baliga NS , Wang JT , Ramage D , Amin N , Schwikowski B , Ideker T . Cytoscape: a software environment for integrated models of biomolecular interaction networks. Genome Res 2003;13(11):2498–504. 10.1101/gr.1239303 14597658 PMC403769

[27] Raman K , Damaraju N , Joshi GK . The organisational structure of protein networks: revisiting the centrality‐lethality hypothesis. Syst Synth Biol. 2014;8(1):73‐81. doi:10.1007/s11693-013-9123-5 24592293 PMC3933631

[28] Zhang Y , Bai M , Zhang B , et al. Uncovering pharmacological mechanisms of Wu‐tou decoction acting on rheumatoid arthritis through systems approaches: drug‐target prediction, network analysis and experimental validation. Sci Rep. 2015;30(5):9463. doi:10.1038/srep09463 Erratum in: Sci Rep 2018 Oct 30;8(1):15924. PMC6207787 30375403

[29] Missiuro PV , Liu K , Zou L , et al. Information flow analysis of interactome networks. PLoS Comput Biol. 2009;5(4):e1000350. doi:10.1371/journal.pcbi.1000350 19503817 PMC2685719

[30] Liu H , Wang L , Lv M , et al. AlzPlatform: an Alzheimer's disease domain‐specific chemogenomics knowledgebase for polypharmacology and target identification research. J Chem Inf Model. 2014;54(4):1050‐1060. doi:10.1021/ci500004h 24597646 PMC4010297

[31] Morris GM , Huey R , Lindstrom W , et al. AutoDock4 and AutoDockTools4: automated docking with selective receptor flexibility. J Comput Chem. 2009;30(16):2785‐2791. doi:10.1002/jcc.21256 19399780 PMC2760638

[32] Trott O , Olson AJ . AutoDock Vina: improving the speed and accuracy of docking with a new scoring function, efficient optimization, and multithreading. J Comput Chem. 2010;31(2):455‐461. doi:10.1002/jcc.21334 19499576 PMC3041641

[33] Wang J , Wong YK , Liao F . What has traditional Chinese medicine delivered for modern medicine? Expert Rev Mol Med. 2018;20:e4. doi:10.1017/erm.2018.3 29747718

[34] Neuschwander‐Tetri BA . Hepatic lipotoxicity and the pathogenesis of nonalcoholic steatohepatitis: the central role of nontriglyceride fatty acid metabolites. Hepatology. 2010;52(2):774‐788. doi:10.1002/hep.23719 20683968

[35] Lewis GF , Carpentier A , Adeli K , Giacca A . Disordered fat storage and mobilization in the pathogenesis of insulin resistance and type 2 diabetes. Endocr Rev. 2002;23(2):201‐229. doi:10.1210/edrv.23.2.0461 11943743

[36] Wiggins D , Gibbons GF . The lipolysis/esterification cycle of hepatic triacylglycerol. Its role in the secretion of very‐low‐density lipoprotein and its response to hormones and sulphonylureas. Biochem J. 1992;284((Pt 2)(Pt 2)):457‐462. doi:10.1042/bj2840457 1599431 PMC1132660

[37] Donnelly KL , Smith CI , Schwarzenberg SJ , Jessurun J , Boldt MD , Parks EJ . Sources of fatty acids stored in liver and secreted via lipoproteins in patients with nonalcoholic fatty liver disease. J Clin Invest. 2005;115(5):1343‐1351. doi:10.1172/JCI23621 15864352 PMC1087172

[38] Ouyang X , Cirillo P , Sautin Y , et al. Fructose consumption as a risk factor for non‐alcoholic fatty liver disease. J Hepatol. 2008;48(6):993‐999. doi:10.1016/j.jhep.2008.02.011 18395287 PMC2423467

[39] Zhang X , Shao H , Zheng X . Amino acids at the intersection of nutrition and insulin sensitivity. Drug Discov Today. 2019;24(4):1038‐1043. doi:10.1016/j.drudis.2019.02.008 30818029

[40] Stahl EP , Dhindsa DS , Lee SK , Sandesara PB , Chalasani NP , Sperling LS . Nonalcoholic fatty liver disease and the heart: JACC State‐of‐the‐Art Review. J Am Coll Cardiol. 2019;73(8):948‐963. doi:10.1016/j.jacc.2018.11.050 30819364

[41] Freeman AM , Pennings N . Insulin resistance. StatPearls [Internet]. StatPearls Publishing; 2021. 29939616

[42] Emma MR , Augello G , Cusimano A , et al. GSK‐3 in liver diseases: friend or foe? Biochim Biophys Acta Mol Cell Res. 2020;1867(9):118743. doi:10.1016/j.bbamcr.2020.118743 32417256

[43] Hughes K , Nikolakaki E , Plyte SE , Totty NF , Woodgett JR . Modulation of the glycogen synthase kinase‐3 family by tyrosine phosphorylation. EMBO J. 1993;12(2):803‐808. 8382613 10.1002/j.1460-2075.1993.tb05715.x PMC413270

[44] Cohen P , Frame S . The renaissance of GSK3. Nat Rev Mol Cell Biol 2001;2(10):769–76. 10.1038/35096075 11584304

